# Editorial on the Special Issue Entitled “Laser Manufacturing and Additive Manufacturing”

**DOI:** 10.3390/ma19040671

**Published:** 2026-02-10

**Authors:** Changjun Chen

**Affiliations:** Laser Processing Research Center, School of Mechanical and Electric Engineering, Soochow University, Suzhou 215131, China; chenchangjun@suda.edu.cn

The pursuit of high-precision, multi-functional, and cost-effective manufacturing technologies has become a core driver of innovation in advanced manufacturing, materials science, and industrial engineering. Laser manufacturing and additive manufacturing (AM) have emerged as transformative pillars in this field, revolutionizing the design and production of complex components across aerospace, automotive, energy, and electronic industries. Laser-based processes, with their superior controllability, high energy density, and non-contact operation, enable precise material processing such as drilling, cutting, deposition, and surface modification. Additive manufacturing, meanwhile, breaks the limitations of traditional subtractive manufacturing by constructing components layer-by-layer, facilitating the fabrication of intricate geometries, multi-material structures, and customized parts with reduced material waste. The integration of these two technologies further amplifies their advantages, opening up new possibilities for producing high-performance components with tailored microstructures and mechanical properties [1].

Despite the remarkable advancements, the widespread industrial adoption of laser manufacturing and additive manufacturing still faces significant challenges. In laser processing, achieving consistent precision in micro-scale operations (e.g., micro-hole drilling) and real-time process monitoring to ensure reliability remain critical hurdles [2]. For additive manufacturing processes such as Laser Powder Bed Fusion (LPBF) and Laser Directed Energy Deposition (LDED), post-processing issues—including the formation of alpha case on high-temperature alloy components, which degrades mechanical performance—pose persistent challenges [3]. Additionally, the fabrication of multi-material structures via integrated AM technologies requires meticulous interface optimization to eliminate defects and ensure stable mechanical and functional synergy between dissimilar materials [4]. The influence of process parameters (e.g., laser energy, tempering treatments) on the microstructure, corrosion resistance, thermal conductivity, and mechanical properties of AM-fabricated alloys and composites also demands in-depth exploration [5]. Furthermore, the development of reliable simulation models to predict process–microstructure–property relationships is essential for reducing experimental costs and accelerating the industrialization of these technologies [6].

To address these critical gaps and showcase the latest research progress, this Special Issue compiles eight original research articles that make significant contributions to the advancement of laser manufacturing and additive manufacturing. These studies cover a broad spectrum of topics, from laser-based processing and AM process optimization to microstructure characterization and performance evaluation, providing valuable insights for both academic research and industrial application.

In the realm of laser manufacturing, one study focuses on laser drilling of micro-holes, proposing a breakthrough detection method to enhance the precision and efficiency of micro-hole fabrication—a key requirement for components in microelectronics and aerospace systems. Another article investigates the effect of laser on the interface and thermal conductivity of metallized diamond/Cu composite coatings deposited by supersonic laser deposition, shedding light on how laser parameters can be tuned to optimize the functional performance of composite coatings for thermal management applications [7].

Regarding additive manufacturing, several studies target process optimization and performance improvement of high-performance alloys. Two identical articles explore the removal of alpha case from LPBF components using cavitation abrasive surface finishing, offering an effective post-processing solution to enhance the mechanical integrity of nickel-based superalloy components. Another study unveils the effect of Ti micro-alloying on the microstructure and corrosion resistance of GH3536 alloy processed by laser metal deposition (LMD) in a simulated proton exchange membrane fuel cell (PEMFC) environment, providing guidance for the development of corrosion-resistant alloys for energy applications [8]. Additionally, one article examines the effects of tempering on the microstructure and properties of AM-fabricated Cu-bearing AISI 431 steel, clarifying the heat treatment mechanism to tailor the mechanical performance of stainless steel components. The interface optimization, microstructural characterization, and mechanical performance of CuCrZr/GH4169 multi-material structures manufactured via LPBF-LDED integrated AM are also investigated, addressing the critical challenge of multi-material integration in advanced manufacturing. Finally, a study combines simulation and experiment to explore the AM of highly dense pure tungsten by LPBF, overcoming the inherent difficulty of fabricating refractory metal components with high density and structural integrity [9].

In summary, the research presented in this Special Issue provides a comprehensive overview of the latest innovations and technical breakthroughs in laser manufacturing and additive manufacturing. These studies not only address key technical bottlenecks but also offer new perspectives for future research directions. Meanwhile, with the care and support of all parties, the first volume has achieved remarkable results. In response to strong demands from numerous readers and authors, we have begun work on the second volume. We warmly welcome and appreciate continued support for our work in advancing the development of laser and additive manufacturing. Moving forward, further progress in this field will rely on the deep integration of experimental and simulation approaches to establish precise process–microstructure–property relationships. The development of intelligent process monitoring and control systems, leveraging technologies such as machine learning and digital twins, will be crucial for improving process stability and reproducibility [10]. Additionally, exploring the potential of multi-material integration, functionalization of AM components, and environmentally sustainable manufacturing processes will expand the application boundaries of laser and additive manufacturing technologies.

We believe this Special Issue will serve as a valuable platform for researchers and engineers to exchange ideas, promote collaborative research, and drive the industrialization of laser manufacturing and additive manufacturing, ultimately contributing to the advancement of global advanced manufacturing industries.
